# Predictors of unplanned hospital readmission after non-cardiac surgery in Singapore: a 2-year retrospective review

**DOI:** 10.1186/s12893-023-02102-7

**Published:** 2023-07-13

**Authors:** Zhao Kai Low, Lydia Liew, Vanessa Chua, Sophia Chew, Lian Kah Ti

**Affiliations:** 1 Department of Anaesthesia, National University Health System, National University Hospital, Main Building, Level 3 (Near Lift Lobby 1), 5 Lower Kent Ridge Road, Singapore, 119074 Singapore; 2 Department of Anaesthesia, Yong Loo Lin School of Medicine, National University of Singapore, Singapore, Singapore; 3 Department of Anaesthesiology, Singapore General Hospital, Singapore, Singapore

**Keywords:** Readmissions, Post-surgery, Asian

## Abstract

**Introduction:**

Unplanned hospital readmissions after surgery contribute significantly to healthcare costs and potential complications. Identifying predictors of readmission is inherently complex and involves an intricate interplay between medical factors, healthcare system factors and sociocultural factors. Therefore, the aim of this study was to elucidate the predictors of readmissions in an Asian surgical patient population.

**Methods:**

A two-year single-institution retrospective cohort study of 2744 patients was performed in a university-affiliated tertiary hospital in Singapore, including patients aged 45 and above undergoing intermediate or high-risk non-cardiac surgery. Unadjusted analysis was first performed, followed by multivariable logistic regression.

**Results:**

Two hundred forty-nine patients (9.1%) had unplanned 30-day readmissions. Significant predictors identified from multivariable analysis include: American Society of Anaesthesiologists (ASA) Classification grades 3 to 5 (adjusted OR 1.51, 95% CI 1.10–2.08, *p* = 0.01), obesity (adjusted OR 1.66, 95% CI 1.18–2.34, *p* = 0.04), asthma (OR 1.70, 95% CI 1.03–2.81, *p* = 0.04), renal disease (OR 2.03, 95% CI 1.41–2.92, *p* < 0.001), malignancy (OR 1.68, 95% CI 1.29–2.37, *p* < 0.001), chronic obstructive pulmonary disease (OR 2.46, 95% CI 1.19–5.11, *p* = 0.02), cerebrovascular disease (OR 1.73, 95% CI 1.17–2.58, *p* < 0.001) and anaemia (OR 1.45, 95% CI 1.07–1.96, *p* = 0.02).

**Conclusion:**

Several significant predictors of unplanned readmissions identified in this Asian surgical population corroborate well with findings from Western studies. Further research will require future prospective studies and development of predictive risk modelling to further address and mitigate this phenomenon.

**Supplementary Information:**

The online version contains supplementary material available at 10.1186/s12893-023-02102-7.

## Introduction

Unplanned hospital readmission rates have been widely regarded as a quality indicator of healthcare services [1], and have been associated with significant healthcare costs, estimated at 15 to 20 billion dollars annually in the United States [2]. This is in addition to numerous other negative consequences including decreased patient satisfaction, prolonged exposure to potential nosocomial infections, as well as increasing demand for hospital beds.

Consequently, identifying predictors of hospital readmissions and reducing readmission rates has now emerged as an important area of research. Prominent risk factors that have been identified from retrospective studies include older patients aged 65 and above, male gender, medical comorbidities including malignancy, renal disease and anaemia, polypharmacy as well as lower socioeconomic status [3, 4]. Several hospital readmission risk prediction models have also been proposed, such as the LACE index and the PARR-30 model [5]. However, the overall predictive ability of these models remains poor [6], and unique sociodemographic variables limit the application of these models in a wider context across different healthcare systems. It is now recognised that hospital readmission prediction is inherently complex, and necessarily involves an intricate interplay between medical factors such as comorbidities and clinical variables, hospital- and healthcare system-specific factors such as access to transitional care facilities, as well as wider social and cultural factors within the population [7].

With the majority of studies thus far centred on Western populations, there is a need to study this in the Asian context, where unique sociocultural factors may influence readmission trends. This is particularly relevant in the setting of the progressively aging population in Asia [8], which will increasingly impose an unprecedented strain on healthcare resources. Understanding the clinical predictors of hospital readmissions specific to the loco-regional context in a predominantly elderly Asian patient population would facilitate further development of predictive models and targeted risk-management strategies to reduce hospital readmissions in the near future. Hence, this study aims to investigate the association of clinical risk factors with 30-day readmissions rates in an Asian surgical population.

## Methodology

Following Institutional Review Board approval, a retrospective cohort study was conducted in a university-affiliated tertiary hospital in Singapore from 2014 to 2015. Inclusion criteria included patients aged 45 years and above undergoing intermediate or high-risk non-cardiac surgery, defined as surgery requiring at least 23 h stay in the hospital. This criterion is based on our hospital’s admission structure, which classifies surgical cases into day cases, 23-h short stay, and ward admissions (> 23 h stay). The majority of day cases are expected to be low-risk patients or minor procedures unlikely to lead to readmissions. Therefore, these patients were not included in our study criteria. In patients who underwent multiple surgeries, only the index surgery was included for analysis.

Relevant demographic, surgical, anaesthetic and readmission data were obtained from patient electronic medical records. Comorbidities were identified from electronic medical records, where they are typically summarised in the admitting documents and discharge summaries. Relevant investigation results were also reviewed, including blood investigations, cardiac investigations and pulmonary function tests. Obesity was defined as a body mass index (BMI) above 27.5, and anaemia was defined as a haemoglobin level below 12.0 g/dL in females and below 13.0 g/dL in males.

Readmissions were defined as hospital readmissions within 30 days of discharge from the index hospitalisation, excluding planned readmissions. Planned and unplanned readmissions were differentiated by checking electronic medical records including ward and clinic notes for any planned readmissions, such as for further investigation or treatment. Unadjusted analysis was first performed using the chi-squared test for categorical variables and t-test for continuous variables. Significant factors identified from the unadjusted analysis with a *p*-value of < 0.1 were further analysed in a single multivariable logistic regression model. Results from the multivariable analysis were considered significant at a *p*-value of < 0.05. All statistical analyses were performed using IBM SPSS v.25.0 [9].

## Results

**Fig. 1:**
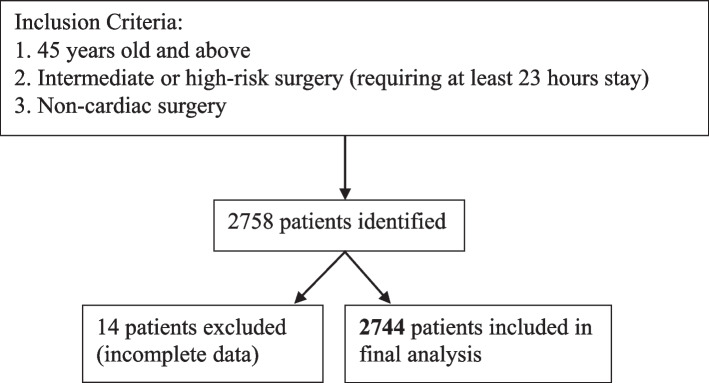
Patient selection

The electronic medical records of 2758 patients were reviewed after fulfilling inclusion criteria. 14 patients had incomplete data and were excluded, leaving a final cohort of 2744 patients (Fig. 1). Patient demographic variables, surgical/anaesthetic characteristics and unadjusted analysis results are presented in Table 1.

**Table 1:** Demographic, surgical and anaesthetic variables, and unadjusted analysis of risk factors associated with 30-day readmission

**Preoperative Risk Factors**	**Not Readmitted** **(*n* = 2495)**	**Readmitted Within 30 Days** **(*n* = 249)**	***p*-value**
Age (years)*	63.3 ± 10.9	65.8 ± 11.0	< 0.001
Gender			0.55
Female	1142 (45.8)	109 (43.8)	
Male	1353 (54.2)	140 (56.2)	
Ethnicity			0.29
Chinese	1749 (70.1)	160 (64.3)	
Malay	483 (19.4)	58 (23.3)	
Indian	207 (8.3)	25 (10.0)	
Others	56 (2.2)	6 (2.4)	
ASA Classification*			< 0.001
ASA 1—2	1326 (54.7)	79 (32.9)	
ASA 3—5	1099 (645.3)	161 (67.1)	
Surgery Status			0.23
Elective	1765 (71.0)	175 (70.3)	
Emergency	722 (29.0)	74 (29.7)	
Surgical Discipline*			0.01
General Surgery, Urology, O&G	993 (39.8)	109 (43.8)	
Orthopaedics	874 (35.0)	70 (28.1)	
Neurosurgery	161 (6.5)	18 (7.2)	
Ophthalmology & Otolaryngology	227 (9.1)	14 (5.6)	
Thoracic and Vascular	181 (7.3)	29 (11.6)	
Others	59 (2.4)	9 (3.6)	
Type of Anaesthesia			0.12
General Anaesthesia	1983 (85.4)	203 (81.5)	
General Anesthesia with CNB	23 (1.0)	6 (2.4)	
Regional Anaesthesia	213 (9.2)	25 (10.0)	
Monitored Anaesthesia Care	102 (4.4)	15 (6.0)	
Duration of Surgery			0.13
Less than 2 h	899 (52.4)	116 (58)	
More than 2 h	818 (47.6)	84 (42)	
Obesity (BMI > 27.5)*	395 (15.8)	59 (23.7)	< 0.001
Smoker	259 (10.4)	25 (10.1)	0.88
Asthma*	134 (5.4)	25 (10.0)	< 0.001
Hypertension*	1454 (58.3)	165 (66.3)	0.02
Hyperlipidaemia*	1209 (48.5)	144 (57.8)	0.01
Diabetes Mellitus*	703 (28.2)	98 (39.4)	< 0.001
Renal Disease*	281 (11.3)	69 (27.7)	< 0.001
Malignancy*	375 (15.0)	57 (22.9)	< 0.001
Congestive Cardiac Failure	59 (2.4)	9 (3.6)	0.23
Ischaemic Heart Disease*	437 (17.5)	67 (26.9)	< 0.001
Valvular Heart Disease	100 (4.0)	13 (5.2)	0.36
Peripheral Vascular Disease*	85 (3.4)	21 (8.4)	< 0.001
Arrhythmias*	150 (6.0)	33 (13.3)	< 0.001
Acute Myocardial Infarction (Within 1 Month)	21 (0.8)	4 (1.6)	0.28
Chronic Obstructive Pulmonary Disease*	37 (1.5)	11 (4.4)	< 0.001
Obstructive Sleep Apnoea	88 (3.5)	12 (4.8)	0.37
Seizures	31 (1.2)	6 (2.4)	0.14
Liver Disease	74 (3.0)	11 (4.4)	0.21
Hematology	92 (3.7)	10 (4.0)	0.79
Cerebrovascular Disease*	242 (9.7)	47 (18.9)	< 0.001
Other CNS Comorbidities*	121 (4.8)	19 (7.6)	0.06
Thyroid Disease	160 (6.4)	18 (7.2)	0.62
Anaemia*^a^	1035 (42.9)	151 (60.9)	< 0.001

Results are presented as n (%) or mean ± standard deviation

*CI* Confidence Interval, *OR* Odds Ratio, *ASA* American Society of Anesthesiologists, *BMI* Body Mass Index, *CNB* Central Neuraxial Blocks, *CNS* Central Nervous System, *O&G* Obstetrics and Gynaecology

^*^Significant factors identified from unadjusted analysis (*p* value < 0.1) which were then further analysed using multivariable logistic regression

^a^Anaemia: Hb ≤ 12.0 g/dL (female), Hb ≤ 13.0 g/dL (male)

The mean age of the study population was 63.5 ± 10.9 years, and 54.4% were male. 69.6% of patients were Chinese, 19.7% were Malay and 8.5% were Indian, which forms a good representation of the ethnic distribution in the Singapore population. Majority of the patients were categorized as American Society of Anesthesiologists (ASA) Classification grades 2 (45.5%) or 3 (40.0%). A large proportion of the surgical caseload comprised of general surgery, obstetrics/gynaecology and orthopaedic surgery cases (74.6% in total), and 85.0% of patients underwent general anaesthesia.

A total of 249 patients (9.1%) had unplanned 30-day hospital readmissions. Significant risk factors identified from multivariable analysis (Table 2) include: ASA Classification grades 3 to 5 (adjusted odds ratio [OR] 1.51, 95% confidence interval [CI] 1.10–2.08, *p* = 0.01), obesity (OR 1.66, 95% CI 1.18–2.34, *p* = 0.04), asthma (OR 1.70, 95% CI 1.03–2.81, *p* = 0.04), renal disease (OR 2.03, 95% CI 1.41–2.92, *p* < 0.001), malignancy (OR 1.68, 95% CI 1.29–2.37, *p* < 0.001), chronic obstructive pulmonary disease (COPD) (OR 2.46, 95% CI 1.19–5.11, *p* = 0.02), cerebrovascular disease (OR 1.73, 95% CI 1.17–2.58, *p* < 0.001) and anaemia (OR 1.45, 95% CI 1.07–1.96, *p *= 0.02).

**Table 2:** Multivariable analysis of risk factors associated with 30-day readmission

	**Multivariable Analysis**		
**Risk Factors**	**OR**	**95% CI**	**Adjusted** ***p*-value**
Age	1.01	1,00 – 1.02	0.14
ASA Classification Grades 3–5	1.51	1.10 – 2.08	0.01
Type of Surgery			
General Surgery, Urology, O&G			
Orthopaedics	0.71	0.50 – 1.00	0.05
Neurosurgery	0.82	0.46 – 1.45	0.49
Ophthalmology & Otolaryngology	0.56	0.30 – 1.04	0.06
Thoracic and Vascular	0.74	0.45 – 1.23	0.24
Others	0.94	0.40 – 2.19	0.88
Obesity (BMI > 27.5)	1.66	1.18 – 2.34	0.04
Asthma	1.70	1.03 – 2.81	0.04
Hypertension	0.86	0.61 – 1.22	0.40
Hyperlipidaemia	1.05	0.76 – 1.46	0.75
Diabetes Mellitus	1.16	0.84 – 1.60	0.36
Renal Disease	2.03	1.41 – 2.92	< 0.001
Malignancy	1.68	1.29 – 2.37	< 0.001
Ischaemic Heart Disease	0.95	0.66 – 1.35	0.76
Peripheral Vascular Disease	1.58	0.89 – 2.79	0.12
Arrhythmias	1.43	0.91 – 2.25	0.12
Chronic Obstructive Pulmonary Disease	2.46	1.19 – 5.11	0.02
Cerebrovascular Disease	1.73	1.17 – 2.58	< 0.001
Other CNS Comorbidities	0.86	0.42 – 1.78	0.70
Anaemia^a^	1.45	1.07 – 1.96	0.02

*CI* Confidence Interval, *OR* Odds Ratio, *ASA* American Society of Anesthesiologists, *BMI* Body Mass Index, *CNS* Central Nervous System, *O&G* Obstetrics and Gynaecology

^a^Anaemia: Hb ≤ 12.0 g/dL (female), Hb ≤ 13.0 g/dL (male)

Other risk factors including age, surgical discipline, hypertension, hyperlipidaemia, diabetes mellitus, ischaemic heart disease, peripheral vascular disease and arrhythmias showed statistical significance in the unadjusted analysis, but were not significant in the multivariable analysis.

## Discussion

There is a need to understand the phenomenon of unplanned post-surgical readmissions in the context of an Asian patient demographic, where unique epidemiological trends, sociocultural elements, as well as healthcare system-related factors will inevitably influence readmission rates and patterns.

For example, within the Singapore healthcare system, there is a tendency towards a longer postoperative length of stay (LOS), which is likely a reflection of underlying sociocultural elements at play. Studies in hip fracture patients have reported a mean LOS of 15.7 days in Singapore [10] compared to 4–5 days in Finland and 8.1 days in the US [11, 12]. A longer duration of index hospital admission has been identified as an independent risk factor for readmissions [13].

### Readmission Rates

The readmission rate of 9.1% in our study population (10.8% in elderly patients above 65 years old) is comparable to that reported in literature. A 2014 systematic review reported median readmission rates of 9.7% and 18.5% in general and vascular surgery patients respectively [14]. Independent studies in Asian patient populations have reported slightly higher 30-day all-cause readmission rates ranging from 13.37% to 15.5% [15, 16]. These studies did not differentiate between medical and surgical patient cohorts.

Two studies focusing on readmission rates in elderly patients above 65 years old have reported a wide range of 30-day all-cause readmission rates, ranging from 7.7% to 19.2% [17, 18]. There are clear differences in patient characteristics which may account for the disparity in readmission rates. In the latter study, close to 30% of patients required assistance with activities of daily living. There is also suggestion that socioeconomic disparities and lack of access to primary healthcare services significantly increase readmission rates in the elderly [19]. Evidently, a complex amalgamation of medical comorbidities, functional status and social factors influence readmission rates, rather than elderly age per se.

### Predictors of 30-Day Unplanned Hospital Readmissions

The significant predictors of 30-day unplanned readmissions identified from multivariable logistic regression include: high ASA grade, obesity, asthma, COPD, renal disease, malignancy, cerebrovascular disease and anaemia. This is consistent with risk factors that have been identified in literature [3, 4]. Among the significant risk factors that have been identified, anaemia is the only potentially modifiable perioperative risk factor, while the others are not modifiable in the perioperative context.

Interestingly, elderly age was not a statistically significant risk factor identified from the multivariable analysis, suggesting that the various increased comorbidities seen with advanced age are the main contributors to the readmission risk, rather than the patient’s age itself. A 2020 retrospective cohort study from our institution [19] reported an adjusted mortality OR of 1.04 for every one-year increase in age, pertaining to one-year perioperative mortality after non-cardiac surgery.

The effect of hospital length of stay on unplanned readmissions was not included in this analysis due to the wide range of surgical conditions studied, which would limit the interpretation of the results. However, in line with studies that have identified LOS as an independent risk factor for hospital readmissions, reducing hospital LOS seems to be a viable target for perioperative optimisation programmes, and can potentially reduce readmission rates. This has been evident in results from Enhanced Recovery after Surgery (ERAS) programmes both locally and internationally, which have reported decreased hospital LOS with similar or decreased readmission rates [20, 21].

### Body Mass Index (BMI)

The association between obesity and surgical readmission rates has been reported in Western surgical populations. In a retrospective case–control study of 1380 readmissions, obese surgical patients were 1.25 times more likely to be readmitted [22]. Meta-analyses in orthopaedic surgical patients have reported obesity as an independent risk factor for surgical site infections and venous thromboembolism [23]. The immunological basis of the pro-inflammatory state seen in obese patients has been attributed to the production of leptin by adipocytes and downstream production of pro-inflammatory cytokines including TNF-alpha, interleukin-6 (IL-6) and IL-12 [24]. The prothrombotic propensity in obesity has been postulated to arise from complex interactions between genetic and environmental factors including interaction with the factor II G2010A mutation in susceptible individuals and pro-inflammatory effects of adipocytokines and free fatty acids leading to mitochondrial production of reactive oxygen species [25].

A lower BMI cut-off value of 27.5 was used in this study in accordance with World Health Organization and American Diabetes Association [26, 27] recommendations for Asian patients, due to a genetic predisposition towards visceral adiposity, higher body fat percentage and increased cardiovascular complications for the same BMI.

Conversely, studies have also demonstrated increased postoperative pulmonary complications and lower survival rates in underweight surgical patients [28, 29]. The catabolic state in these patients with associated immunologic suppression, poor wound healing and decreased functional reserve contribute to increased postoperative infective complications. This can in turn lead to delayed discharge, higher readmissions and potentially delayed commencement of adjuvant therapies after oncological resections. Consequently, preoperative and early postoperative nutritional assessment and optimisation protocols are an essential component in the multidisciplinary care of surgical patients, particularly in underweight patients at risk of malnutrition and catabolism.

### Asthma and Chronic Obstructive Pulmonary Disease (COPD)

Patients with asthma or COPD had a two-fold higher readmission rate in our study population. Asthma and COPD have been shown to be independent risk factors for postoperative pulmonary complications (including pneumonia, bronchospasm and pulmonary embolism) and 30-day postoperative mortality [30, 31]. Among these patients, specific factors associated with postoperative pulmonary complications include upper abdominal surgery and long operating time (> 5 h). Beyond the pulmonary pathophysiology of asthma and COPD leading to expiratory airflow limitation, atelectasis and ventilation-perfusion mismatch, the extrapulmonary effects of the disease likely also contribute significantly to adverse perioperative outcomes. These include the systemic inflammatory state associated with COPD leading to catabolic weight loss, skeletal muscle dysfunction and osteoporosis [32], as well as accelerated atherosclerosis which forms an independent risk factor for cardiovascular and cerebrovascular disease in COPD patients [33].

Perioperative interventions including pulmonary rehabilitation, as well as modifications in anaesthetic and surgical techniques have shown promise in reducing complication rates, and should be routinely considered while preparing the asthma or COPD patient for surgery. A US registry-based retrospective study involving 2644 propensity-matched surgical patients reported reduced postoperative pulmonary complications with regional anaesthetic techniques [34]. A meta-analysis of COPD patients undergoing major gastrointestinal surgery reported a significantly lower rate of postoperative pneumonia with the laparoscopic approach [35]. The surgical and anaesthetic approach to the asthma or COPD patient should be risk-stratified and tailored according to the patient’s disease severity, extent of surgery, urgency of operation and time available for preoperative optimisation. Postoperative care and patient education by a specialised asthma/COPD practitioner and discharge planning are also equally important in optimising outcomes and reducing readmissions.

### Renal Disease

Both acute and chronic renal disease/end-stage renal failure have been linked with increased readmission rates, and the risk appears to be commensurate with the severity of renal impairment [36]. Common causes of readmission in renal patients include vascular access revision, volume overload and anaemia [37], especially with haematocrit levels below 30% [38]. Conversely, protective factors that have been identified include serum albumin levels above 35 g/L [39], anaemia management programmes [40], as well as early outpatient nephrology review within one month of discharge [41]. Reducing readmission rates in renal patients requires holistic management of the multisystemic effects of renal disease and a multidisciplinary approach to discharge planning. Possible strategies include timely inpatient nephrology consult and dialysis support, early outpatient nephrology review, anaemia and volume management protocols, medication reconciliation and patient education, nutrition optimisation, as well as comprehensive discharge planning to ensure adequate social support and continuity of care at outpatient dialysis centres.

### Anaemia

The association between perioperative anaemia and adverse postoperative outcomes has been well-studied. A US registry-based analysis of 227,425 patients undergoing major non-cardiac surgery reported higher 30-day postoperative morbidity and mortality rates in anaemic patients, which was consistent across patients with mild anaemia and moderate-to-severe anaemia [42]. While anaemia may often reflect underlying chronic conditions and potential confounders, our results support its role as an independent risk factor, and underline the importance of perioperative anaemia management strategies to optimise postoperative outcomes.

A 3-year single-institution study investigating the effects of a preoperative anaemia management protocol in cardiac surgical patients reported reduced transfusions, fewer days in the intensive care unit (ICU), shorter LOS, as well as significant cost savings, although readmission rates were not reported [43]. Beyond the beneficial effects on myocardial oxygen supply and tissue oxygen delivery, preoperative treatment of anaemia would importantly reduce the need for perioperative transfusions with its associated risks such as infections, circulatory overload and transfusion-related immunomodulation.

### Malignancy

Patients with malignancy had an almost two-fold increased readmission risk in our study population. Readmissions in cancer patients have been associated with poorer survival rates in several large-scale registry-based retrospective studies in patients who underwent surgical resections of colon cancer, lung cancer and pancreatic cancer [44–46]. Common causes of early readmissions include gastrointestinal, respiratory and infective complications, while late readmissions were related to metastatic disease and thromboembolic complications.

While this association does not prove causality and may in some cases reflect the appropriate readmission of patients suffering from severe or progressive disease, there are certainly deleterious effects of hospital readmission and prolonged stay in this vulnerable patient group, including increased exposure to nosocomial infections, propensity for functional decline, and potentially delayed commencement of adjuvant cancer therapy.

Furthermore, it is estimated that a significant proportion of these readmissions may be potentially preventable. In a US-based analysis of 59,493 registry patients undergoing complex cancer surgery, 14% of patients were readmitted within 30 days, of which 82% were classified as potentially preventable readmissions (PPRs) that could be reasonably prevented with improved provision of quality inpatient care, discharge planning, or post-discharge follow-up [47].

### Cerebrovascular Disease

Patients with a history of cerebrovascular disease were 1.7 times more likely to experience an unplanned readmission. There was no information regarding the neurological condition and functional status of these patients, which may provide an important clue regarding this elevated readmission risk. Functional disability and decreased activities of daily living at discharge were shown to significantly increase readmission and mortality rates in a single-centre Japanese retrospective study [48]. Additionally, sociocultural factors can be expected to significantly shape this phenomenon, including socioeconomic status, availability of social and family support, and access to primary care and community resources.

### Impact of COVID-19 Pandemic

The phenomenon of hospital readmissions driving demand for healthcare resources is expected to be further compounded by the COVID-19 pandemic, with possible future new strains and resurgences contributing to demand for hospital beds. The impact of the pandemic on unplanned readmissions after surgery is unknown as elective surgical volumes have decreased in the last 3 years across centres worldwide. While it has now been suggested that elective surgeries may be safely continued during the pandemic [49], concerns regarding intra-hospital COVID transmission have resulted in shorter post-surgical length of stay, without increases in readmission rates [50].

## Conclusion

This retrospective study of clinical predictors of unplanned hospital readmissions in an Asian surgical population corroborates well with findings from major studies in Western populations.

The limitations of a retrospective study should be considered in the interpretation of the study results, and we are unable to determine causality within the limits of a retrospective analysis. Future prospective studies are needed to validate these findings, and to determine the efficacy of potential interventions to reduce readmission rates.

The phenomenon of hospital readmissions is inherently complex and multifaceted. Beyond the individual patient’s comorbidities and clinical risk factors, sociocultural elements and wider healthcare system-level factors invariably influence the readmission risk. The future development of predictive modelling and targeted risk management strategies must necessarily incorporate all of these factors, in order to be clinically relevant and applicable to a uniquely Asian surgical patient population.

## Supplementary Information

**Additional file 1: Supplementary Table 1. **Subgroup unadjusted analysis of risk factors associated with 30-day readmission in patients aged 65 years and above.

## Data Availability

All data generated or analysed during this study are included in this published article [and its supplementary information files].
