# Supplementary material for: Predictors of unplanned hospital readmission after non-cardiac surgery in Singapore: a 2-year retrospective review
Source: BMC Surg. 2023 Jul 13;23:202. doi: 10.1186/s12893-023-02102-7 (PMC10347775; doi:10.1186/s12893-023-02102-7)
Supplement: Supplementary file 1 — Additional file 1: Supplementary Table 1. Subgroup unadjusted analysis of risk factors associated with 30-day readmission in patients aged 65 years and above. [file 12893_2023_2102_MOESM1_ESM.docx]

**Supplementary Table 1: Subgroup Unadjusted Analysis of Risk Factors Associated With 30-Day Readmission in Patients Aged 65 Years And Above**

| **Preoperative Risk Factors** | **Not Readmitted**  **(n = 1044)** | **Readmitted Within 30 Days**  **(n = 127)** | **OR** | **95% CI** | **p-value** |
| --- | --- | --- | --- | --- | --- |
|  |  |  |  |  |  |
| Age (years) | 73.7 ± 7.3 | 74.5 ± 7.1 | 1.01 | 0.99 – 1.04 | 0.28 |
| Gender |  |  |  |  | 0.35 |
| Female | 491 (47.0) | 54 (42.5) |  |  |  |
| Male | 553 (53.0) | 73 (57.5) | 1.20 | 0.83 – 1.74 |  |
| Ethnicity |  |  |  |  | 0.77 |
| Chinese | 824 (78.9) | 100 (78.7) |  |  |  |
| Malay | 132 (12.6) | 15 (11.8) | 0.94 | 0.53 – 1.66 |  |
| Indian | 71 (6.8) | 11 (8.7) | 1.28 | 0.65 – 2.49 |  |
| Others | 17 (1.6) | 0 (0.0) | 0.49 | 0.06 – 3.68 |  |
| Obesity (BMI > 27.5) | 133 (12.7) | 20 (15.7) | 1.28 | 0.77 – 2.13 | 0.34 |
| Smoker | 82 (7.9) | 12 (9.5) | 1.24 | 0.65 – 2.33 | 0.52 |
| Asthma | 55 (5.3) | 10 (7.9) | 1.54 | 0.76 – 3.10 | 0.27 |
| Hypertension | 747 (71.6) | 93 (73.2) | 1.09 | 0.72 – 1.65 | 0.69 |
| Hyperlipidaemia | 620 (59.4) | 80 (63.0) | 1.16 | 0.80 – 1.70 | 0.43 |
| Diabetes Mellitus | 329 (31.5) | 46 (36.2) | 1.23 | 0.84 – 1.81 | 0.28 |
| Renal Disease* | 141 (13.5) | 35 (27.6) | 2.44 | 1.59 – 3.74 | <0.001 |
| Malignancy* | 176 (16.9) | 30 (23.6) | 1.53 | 0.98 – 2.37 | 0.06 |
| CCF | 36 (3.4) | 3 (2.4) | 0.68 | 0.21 – 2.23 | 0.79 |
| IHD* | 240 (23.0) | 39 (30.7) | 1.49 | 0.99 – 2.22 | 0.05 |
| VHD | 54 (5.2) | 9 (7.1) | 1.40 | 0.67 – 2.91 | 0.37 |
| PVD* | 42 (4.0) | 13 (10.2) | 2.72 | 1.42 – 5.22 | 0.002 |
| Arrhythmias | 100 (9.6) | 18 (14.2) | 1.56 | 0.91 – 2.67 | 0.10 |
| AMI (Within 1 Month) | 16 (1.5) | 1 (0.8) | 0.51 | 0.07 – 3.87 | >0.99 |
| COPD* | 32 (3.1) | 8 (6.3) | 2.13 | 0.96 – 4.72 | 0.07 |
| OSA | 21 (2.0) | 3 (2.4) | 1.18 | 0.35 – 4.01 | 0.74 |
| Seizures* | 7 (0.7) | 4 (3.1) | 4.82 | 1.39 – 16.69 | 0.02 |
| Liver Disease | 28 (2.7) | 5 (3.9) | 1.49 | 0.56 – 3.92 | 0.39 |
| Hematology | 47 (4.5) | 6 (4.7) | 1.05 | 0.44 – 2.51 | 0.91 |
| CVA* | 140 (13.4) | 36 (28.3) | 2.55 | 1.67 – 3.91 | <0.001 |
| Other CNS Comorbidities | 61 (5.8) | 12 (9.4) | 1.68 | 0.88 – 3.22 | 0.11 |
| Thyroid | 68 (6.5) | 12 (9.4) | 1.50 | 0.79 – 2.85 | 0.26 |
| Anaemia*^†^ | 498 (48.7) | 76 (59.8) | 1.57 | 1.08 – 2.29 | 0.02 |

Results are presented as n (%) or mean ± standard deviation

*Significant factors identified from unadjusted analysis (p value <0.1) which were then further analysed using multivariable logistic regression

CI: Confidence Interval; OR: Odds Ratio

AMI: Acute Myocardial Infarction; BMI: Body Mass Index; CCF: Congestive Cardiac Failure; CNS: Central Nervous System; COPD: Chronic Obstructive Pulmonary Disease; CVA: Cerebrovascular Accident; IHD: Ischaemic Heart Disease; OSA: Obstructive Sleep Apnoea; PVD: Peripheral Vascular Disease; VHD: Valvular Heart Disease

^†^ Anaemia: Hb ≤12.0 g/dL (female), Hb ≤13.0 g/dL (male)
